# First Draft Genome Sequence of the *Acidovorax caeni* sp. nov. Type Strain R-24608 (DSM 19327)

**DOI:** 10.1128/genomeA.01378-15

**Published:** 2015-11-19

**Authors:** Elham Ehsani, Ruy Jauregui, Robert Geffers, Michael Jarek, Nico Boon, Dietmar H. Pieper, Ramiro Vilchez-Vargas

**Affiliations:** a Laboratory of Microbial Ecology and Technology(LabMET), University of Ghent, Ghent, Belgium; b Microbial Interactions and Processes Research Group, Helmholtz Centre for Infection Research, Braunschweig, Germany; c Genome Analytics, Helmholtz Centre for Infection Research, Braunschweig, Germany

## Abstract

We report the draft genome sequence of the *Acidovorax caeni* type strain R-24608 that was isolated from activated sludge of an aerobic-anaerobic wastewater treatment plant. The closest strain to *Acidovorax caeni* strain R-24608 is *Acidovorax* sp. strain MR-S7 with a 55.4% (amino-acid sequence) open reading frames (ORFs) average similarity.

## GENOME ANNOUNCEMENT

*Acidovorax* isolates (*Comamonadaceae* family) are Gram-negative, aerobic bacteria with a polar flagellum and 62 to 70% G+C (1). *Acidovorax* species have been isolated from soil or water (1, 2), infected plants (3, 4), activated sludge (5–7), and clinical samples (1, 8–10). *Acidovorax radicis* N35 was identified as a root-colonizing bacterium without phytopathogenic potential, which showed plant-growth-promoting activity (11). Autotrophic growth with hydrogen as an electron donor was found in *Acidovorax facilis* and some strains of *Acidovorax delafieldii* (1). Denitrification in wastewater treatment plants has been proposed to be catalyzed by members of the *Acidovorax* genus (6, 12, 13) and based on 16S rRNA gene sequence analysis, 19 denitrifiers were recently identified as *Acidovorax avenae*, *A. defluvii*, and *A. temperans* species (5).

Four novel denitrifyers were characterized as belonging to a new species termed *Acidovorax caeni* sp. nov. Here, we announce the first draft genome sequence of the *Acidovorax caeni* sp. nov. type strain R-24608 (14).

The genome was sequenced using the Illumina MiSeq platform, which generated pair-end read sequences of 250 bp. Assemblage using Edena (15, 16) produced 152 contigs with a total genome size of 4,190,928 bs (63.2% G+C content, *N*_50_: 78.99 Kbp, mean: 27.43 Kb) and an average of 83.9-fold coverage. Automatic annotation was performed using the RAST server version 4.0 (17), generating 3,904 features potentially assigned to protein-encoding genes (open reading frames [ORFs]).

A comparison between the draft genome of *Acidovorax caeni* strain R-24608 and the 17 genomes or draft genomes currently available for *Acidovorax* isolates, *Acidovorax* sp. JHL-3 (PRJNA195669), JHL-9 (PRJNA195668), NO-1 (18), CF 316 (19), MR-S7 (20), JS42 (PRJNA15685), KKS102 (21) and *A. citrulli* AAC00-1 (PRJNA15708), *A. citrulli* ZJU1106 (PRJNA175738), *A. avenae* ATCC 19860 (PRJNA37867), *A. avenae* RS-1 (22), *A. radices* N35 (PRJNA64465), *A. radices* N35v (PRJNA64471), *A. ebreus* TPSY (23), *A. delafieldii* 2AN (PRJNA32605), *A. oryzae* ATCC 19882 (PRJNA223028), and *A. temperans* KY4 (PRJNA270288) indicated *Acidovorax* sp. strain MR-S7 with a 55.4% (amino-acid sequence) of ORFs average similarity to be the most closely related to strain R-24608. Both strains share 1,318 ORFs with >80% similarity and 934 ORFs observed in the genome of R-24608 were absent in the genome of the strain MR-S7.

Genes involved in denitrification are encoded by nitrate respiration (*nar*), nitrite respiration (*nir*), nitric oxide respiration (nor), and nitrous oxide respiration (*nos*) genes (24). Strain R-24608 harbors the operon *nar*GHJI and upstream two *nar*K genes encoding nitrate transporter proteins as previously described (25). Nitrite respiration, encoded by the *nir*SCFDLHJN operon was found in R-24608 and we observed that this genomic organization was spread in most of the *Acidovorax* species. The operon involved in nitric oxide respiration, *nor*EFCBQD, was identified downstream from the *nir* operon as previously observed in *Brucella suis* 1330 (26). Nitrous oxide respiration, encoded by the *nos*DFYL operon, was located upstream of the *nos*R gene and the catabolic subunit *nos*Z was eliminated as previously found in *Neisseria meningitidis* MC58 (27).

### Nucleotide sequence accession numbers.

This draft genome sequencing project has been deposited at the EMBL-EBI European Nucleotide Archive under accession numbers CYIG0100000001 to CYIG0100000152.
